# Extraneous E-Cadherin Engages the Deterministic Process of Somatic Reprogramming through Modulating STAT3 and Erk1/2 Activity

**DOI:** 10.3390/cells10020284

**Published:** 2021-01-31

**Authors:** Yu-Hao Liu, Chien-Chang Chen, Yi-Jen Hsueh, Li-Man Hung, David Hui-Kang Ma, Hung-Chi Chen, Wen-Bin Len, Yaa-Jyuhn J. Meir

**Affiliations:** 1 Graduate Institute of Biomedical Sciences, College of Medicine, Chang Gung University, Taoyuan 33302, Taiwan; jxxxaf@gmail.com (Y.-H.L.); oscar11083909@gmail.com (C.-C.C.); lisahung@mail.cgu.edu.tw (L.-M.H.); 2 Center for Tissue Engineering, Chang Gung Memorial Hospital, Linkou 33305, Taiwan; t6612@seed.net.tw (Y.-J.H.); davidhkma@yahoo.com (D.H.-K.M.); mr3756@cgmh.org.tw (H.-C.C.); 3 Limbal Stem Cell Laboratory, Department of Ophthalmology, Chang Gung Memorial Hospital, Linkou 33305, Taiwan; 4 Department of Biomedical Sciences, College of Medicine, Chang Gung University, Taoyuan 33302, Taiwan; 5 Kidney Research Center, Chang Gung Memorial Hospital, Linkou 33305, Taiwan; 6 Department of Chinese Medicine, College of Medicine, Chang Gung University, Taoyuan 33302, Taiwan; 7 Department of Medicine, College of Medicine, Chang Gung University, Taoyuan 33302, Taiwan; 8 Department of Nursing, Chang Gung University of Science and Technology, Taoyuan 33302, Taiwan

**Keywords:** induced pluripotent stem cell (iPSC), somatic reprogramming, Col1a1 4F2A Oct4-GFP, reprogrammable mouse, stochastic and deterministic model

## Abstract

Although several modes of reprogramming have been reported in different cell types during iPSC induction, the molecular mechanism regarding the selection of different modes of action is still mostly unknown. The present study examined the molecular events that participate in the selection of such processes at the onset of somatic reprogramming. The activity of STAT3 versus that of Erk1/2 reversibly determines the reprogramming mode entered; a lower activity ratio favors the deterministic process and vice versa. Additionally, extraneous E-cadherin facilitates the early events of somatic reprogramming, potentially by stabilizing the LIF/gp130 and EGFR/ErbB2 complexes to promote entry into the deterministic process. Our current findings demonstrated that manipulating the pSTAT3/pErk1/2 activity ratio in the surrounding milieu can drive different modes of action toward either the deterministic or the stochastic process in the context of OSKM-mediated somatic reprogramming.

## 1. Introduction

Somatic reprogramming is a process that allows a highly differentiated cell to regain pluripotency by restoring a highly differentiated epigenetic landscape to the pluripotent configuration. Such an event allows the remodeled genome to regain the ability to adopt various cell fates. Previous works have categorized such extraordinarily dynamic and complicated processes into three major phases, namely, initiation, maturation, and stabilization [1]. Most of the previously identified molecules participate in shaping the epigenetic landscape at the transcriptional level and form the genetic circuitry during the maturation and stabilization stages. After reaching the naïve pluripotent state, external stimuli are no longer required for self-renewal. The process relays the progressively remodeling genetic circuitries to the self-activated innate program, where a balance of lineage specifiers has been established, as demonstrated in embryonic stem cells [2,3]. Even with those substantial findings, the molecular mechanism responsible for path selection during the initial process of reprogramming is still poorly understood. Accordingly, several intriguing questions have been raised. For example, what are the criteria for a cell to be selected as an iPSC candidate from a highly differentiated population? What is the role of its surrounding microenvironment? Moreover, how is the milieu information transduced to induce somatic reprogramming?

While various differentiated cell types have been adopted as the cell source for reprogramming, several action modes of somatic reprogramming have been observed, including the stochastic, deterministic, early stochastic and late deterministic, and biphasic models [4,5]. Those modes of action are discernable by the timing of phase progression and the adopted analytic approach during epigenetic remodeling. Among these various reprogramming models, the stochastic process is the most common reprogramming procedure observed in MEF-based reprogramming (MEF; mouse embryonic fibroblast). As the stochastic model posits that all candidates have an equal chance of undergoing reprogramming after certain cell divisions, the acquisition of pluripotency is, therefore, a random event. Currently, the consensus regarding the stochastic model mainly consists of OSKM-mediated somatic reprogramming under the MEF cell context (OSKM; the coding of Oct4, Sox2, Klf4 & cMyc genes linked by the 2A sequences) [5]. Within such transgene-mediated reprogramming, further genetic manipulations are amenable to accelerate the kinetics of such a mode of action through increasing cell proliferation or enhancing the cell-intrinsic mechanisms observed in the clonal analysis of single B cells [6].

Nevertheless, evidence of the deterministic model was also observed in a subpopulation of fast-cycling bone marrow cells. Similarly, removing the roadblocks through genetic manipulations by overexpressing C/EBPα or depleting Mbd3/NurD can produce such a pattern as well [7,8,9]. Unlike the stochastic model, in which each cell has an equal opportunity for reprogramming, only specific cells with a privileged state can overcome the epigenetic barriers in the deterministic process. The deterministic model of reprogramming, therefore, is a synchronized method to surpass the hurdle of the mesenchymal-to-epithelial transition (MET) [8,9]. Although each mode of action has strongly supportive evidence, the molecular mechanisms regarding the process of each mode entry, as well as the subsequent route choice, are still mostly unknown.

In the present study, we adopted MEF-based somatic reprogramming to study the entry mode choice of reprogramming. MEF cells derived from the Col1a1 4F2A Oct4-GFP triple transgenic mouse contain only two copies of OSKM transgenes inserted in the Col1A1 loci in the homozygous animal. In the presence of doxycycline, MEF^Col1a1 4F2A Oct4-GFP^ cells will undergo somatic reprogramming and avoid the potential positional effect and copy number variation inherent in vector-mediated approaches. Under such an inducible OSKM context, reprogramming acts in a stochastic manner in the presence of doxycycline alone. However, somatic reprogramming switches to the deterministic process when extraneous E-cadherin recombinant protein (NTF1) is provided.

Interestingly, this effect is mediated through the prior recognized EGFR/Erb2 and LIFR/GP130 pathways, which function in the late reprogramming transition states between the pre-iPSC, primed iPSC, and naïve iPSC maintenance states [10,11,12]. Additionally, the deterministic model adoption observed due to NTF1-mediated signal transduction results from the output ratio of pSTAT3 and pErk1/2. By manipulating the activity of pSTAT3 and pErk1/2, the mode of action of somatic reprogramming in MEF^Col1a1 4F2A Oct4-GFP^ cells becomes interchangeable. Therefore, the selection of the mode of entry for somatic reprogramming may mainly depend on the differential outputs of pSTAT3 and pErk1/2 if they are not the only determinant factors.

## 2. Materials and Methods

### 2.1. Mouse Embryonic Fibroblast Isolation

All the animal breeding and manipulations followed the care and use of experimental animal guidelines and approved by IACUC, CGU. E13.5 mouse embryos derived from homozygous Col1a1 4F2A Oct4-GFP mice were harvested. After removing the head and major organs, the remaining part of the body was rinsed three times in HBSS. After rinsing, the bodies were minced with scissors and treated with 1× papain at 37 °C for 20 min. Cells were then rinsed with HBSS three times and resuspended in MEF medium (H-D DMEM with 10% FBS, 10 mM NEAA, 10 mM HEPES, 1 mM L-glutamine, 100 units/mL penicillin, and 100 µg/mL streptomycin). Cells were cultured on 10-cm dishes at 37 °C with 5% CO_2_.

### 2.2. iPSC Induction and Colony Counting

MEF^Colla1 4F2A-Oct4-GFP^ cells were maintained in MEF medium before induction. During the process of iPSC induction, 1 × 10^4^ MEF^Colla1 4F2A-Oct4-GFP^ cells were seeded with γ-irradiated feeder cells in one well of a 6-well plate. Twenty-four hours later, doxycycline (1.5 µg/mL) was added to the iSF medium to activate the expression of Yamanaka factors [13]. The induction medium was changed each day during the entire induction process. The Oct4-GFP positive clones were counted from the 7th to 21st days postdoxycycline induction to determine the efficiency of reprogramming.

### 2.3. Protein Analysis

The cell lysate was subjected to 13,000× *g* centrifugation for 15 min at 4 °C to remove the insoluble fraction (CytoBluster protein extraction reagent, Novagen, Madison, WI, USA). The collected supernatant was subjected to SDS-PAGE and transferred to a PVDF membrane. The blotted membranes were incubated with primary antibody overnight, as indicated in each figure. The antibodies included anti-pSTAT3 (CST #9131, 1:1000), anti-STAT3 (SC-482, 1:2000), anti-pErk1/2 (CST#9101, 1:1000), anti-Erk1/2 (CST#9102, 1:1000), anti-pAkt (CST#4060, 1:1000), anti-E-cadherin (BD610182, 1:3000), anti-MYC (Sc-40, 1:2000) and anti-α-tubulin (SC-32293, 1:2000). Then, the blotted membranes were rinsed with TBST before incubating with the secondary antibody at room temperature for 1 h. The secondary antibodies used in the present study included horseradish peroxidase (HRP)-conjugated goat anti-mouse and anti-rabbit IgG. The membrane was rinsed three times by TBST before applying the ECL substrate to visualize the signal.

### 2.4. RNA Extraction and Quantitative PCR Analysis

The RNA isolation method was adopted from the standard TRizol extraction procedure (Molecular Cloning, the 3rd edition). After quantifying the concentration of extracted RNA, 2.5 µg of total RNA was used to produce cDNA. Real-time qPCR (Waltham, MA, USA) with ABI Master Mix was used to quantify gene expression. The primer sets for each studied gene are listed in Table S1.

### 2.5. Construction of Recombinant NTF1 and Protein Purification

The MYC-His6 tag was used to replace the GFP coding sequence from the pCMV-CDH-NTF1-GFP through the SacII/NotI restriction sites to build the NTF1-MYC-His6 fusion protein. The resulting plasmid, pCMV-CDH-NTF1-MYC-His6, was transfected into Freestyle 293 cells with the PEI reagent (Polysciences, Warrington, PA, USA; 23966-1). At 72 h after transfection, the collected medium was subjected to an Ni-resin bead-filled column for NTF1-MYC fusion protein purification. The BSA standard was used to estimate the final concentration of NTF1.

### 2.6. Phosphor-Kinase Array and Receptor Tyrosine Kinase Array

After serum starvation for 24 h, MEF^Col1a1 4F2A Oct4-GFP^ cells were treated with 1 µg/mL NTF1 for 120 or 150 min. Following the manufacturer’s instructions, 400 µg of total protein extract was applied to each blot and then incubated overnight. The visualization steps followed the manufacturer’s instructions (R&D systems, Minneapolis, MN, USA; ARY003B, and ARY014).

### 2.7. NTF1 Treatment and Use of Inhibitors

MEF^Col1a1 4F2A Oct4-GFP^ cells were subjected to 24 h of serum starvation followed by NTF1 treatment. The working concentrations of the four small molecular inhibitors used in the present study were as follows: Gefitinib, 10 μM; Lapatinib, 10 μM; PD0325901, 1 μM; WP1066, 10 μM. Regarding the application of inhibitors, cells were first treated with inhibitors for 15 min before NTF1 treatment.

Similarly, to examine the effect of the DECMA-1 antibody on NTF1 treatment, MEF cells were treated with NTF1, HAV peptide, HGV peptide, or DECMA-1 after 24-h serum starvation. After 2 h of treatment, cells were harvested and lysed for western blotting using anti-pSTAT3 (Y705), -STAT3, -pErk, and -Erk antibodies. The working concentrations of DECMA-1 (ThermoFisher Scientific, Waltham, MA, USA; 14-3249-82), HAV, and HGV peptide were 5 µg/mL, 5 mM, and 5 mM, respectively.

### 2.8. Teratoma Formation Assays

Isolated iPSCs^Col1a1 4F2A Oct4-GFP^ were cultured with γMEF^DR4^ feeder cells in R1 ESC medium. Then, 1.5 × 10^6^ cells were mixed with Matrigel and subcutaneously injected into the flank of 4–6-week-old CB17-SCID mice to produce teratomas. The development of teratomas usually required 14–21 days. Harvested teratomas with a diameter of 1.5 cm were subjected to H&E staining and pathological examination.

### 2.9. Generating the SNL-CDH1-MYC Cell Lines

**Figure 1:**
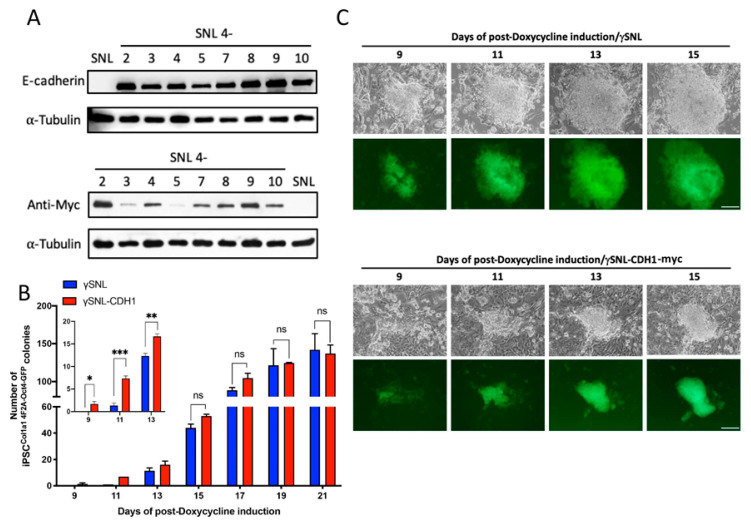
E-cadherin produces in trans facilitation of early-stage somatic reprogramming. (**A**) The expression of E-cadherin in different engineered SNL-CDH1-MYC cell lines. Each SNL-CDH1-MYC line was subjected to western blot analysis with anti-E-cadherin and anti-MYC antibodies. (**B**) Quantitative analysis of the Oct4-GFP-positive colonies during iPSC induction (n = 3). (**C**) Time-lapse photographs of iPSC colony formation from either γSNL or γSNL-CDH1-MYC feeder cell-seeded MEF^Col1a1 4F2A Oct4-GFP^ cells in iSF medium. Fluorescence microscopy was performed on the 9th to 15th days. The iPS clones in the upper panel were seeded with the parental SNL feeder cells, whereas the lower panel shows clones with the SNL-CDH1-MYC feeder cells. The scale bar denotes 50 µm. *, *p* < 0.05; **, *p* < 0.005; ***, *p* < 0.0005; ns, not significant.

The coding of CDH1 was dropped into the pCAG-MCS-MYC-FMDVires-hygroPA vector through SfiI and NotI restriction sites to produce the pCAG-CDH1-MYC-FMDVires-hygroPA vector. After that, we transfected the pCAG-CDH1-MYC-FMDVires-hygroPA plasmid into SNL cells (mouse fibroblast STO cell line transformed with neomycin resistance and murine LIF genes) via the lipofectamine^TM^ (ThermoFisher Scientific, Waltham, MA, USA). Eight hygromycin resistant clones were clonally isolated and cryopreserved. To investigate the CDH1 expression, we conducted western blots using antibodies against CDH1 and MYC, respectively, as shown in Figure 1A.

## 3. Results

### 3.1. E-Cadherin Obtained from SNL Feeder Cells (in Trans) Promotes the Early Stage of Somatic Reprogramming

In addition to promoting cell-cell adhesion, the homophilic interaction between E-cadherin (CDH1) molecules is also essential for the maintenance of pluripotency [14,15]. Prior reports also demonstrated that overexpression of CDH1 in highly differentiated MEFs can replace the requirement of OCT4 during the process of somatic reprogramming [14]. To determine whether providing CDH1 from the surrounding environment benefits iPSC formation, we engineered and selected SNL cells that stably expressed CDH1-MYC. As shown in Figure 1A, both anti-CDH1 and anti-MYC antibodies were used to determine the protein expression level. Eight clonally isolated SNL::CDH1-MYC lines with various expression levels of CDH1-MYC were harvested. Among these eight clones, the SNL4-2 clone displayed the highest expression level of the CHD1-MYC fusion protein, whereas the parental SNL cell line served as a control because we failed to detect the expression of endogenous CDH1. Hereafter, we adopted the SNL4-2 clone to serve as a feeder for the following iPSC induction experiments.

To determine whether CHD1 supplied in trans facilitates the process of somatic reprogramming, 1 × 10^4^ MEF^Col1a1 4F2A Oct4-GFP^ cells were seeded with gamma-irradiated SNL4-2 (γSNL4-2) and parental SNL cells in the presence of doxycycline to trigger expression of the Yamanaka factor. The iPSC^Col1a1 4F2A Oct4-GFP^ colonies produced were counted every other day from the 7th to 21st day postdoxycycline induction. The OCT4-GFP signal served as a criterion for pluripotency acquisition and was used to identify colonies. As shown in Figure 1B, the capability to form iPSCs was not significantly different between SNL::CDH1-MYC and SNL parental feeder cells during the 21-day induction course. Additionally, the morphology and growth rate of the iPSC^Col1a1 4F2A Oct4-GFP^ clones was not affected by the extra E-cadherin support from the feeders (Figure 1C). However, an apparent difference regarding the colony-forming ability was observed at approximately the 9th and 11th days postdoxycycline induction in a triplicate experiment, as shown in the inset of Figure 1B. A similar effect was also observed when SNL4-8 feeder cells, another independently isolated SNL::CDH1-MYC clone, were adopted (data not shown). Therefore, the microenvironment created by the SNL::CDH1-MYC cells does improve the early stage of somatic reprogramming.

### 3.2. Extraneous Recombinant E-cadherin Protein (NTF1) Modulates MEF Cell Status at the Onset of iPSC Formation Resulting in Adoption of the Deterministic Process

**Figure 2:**
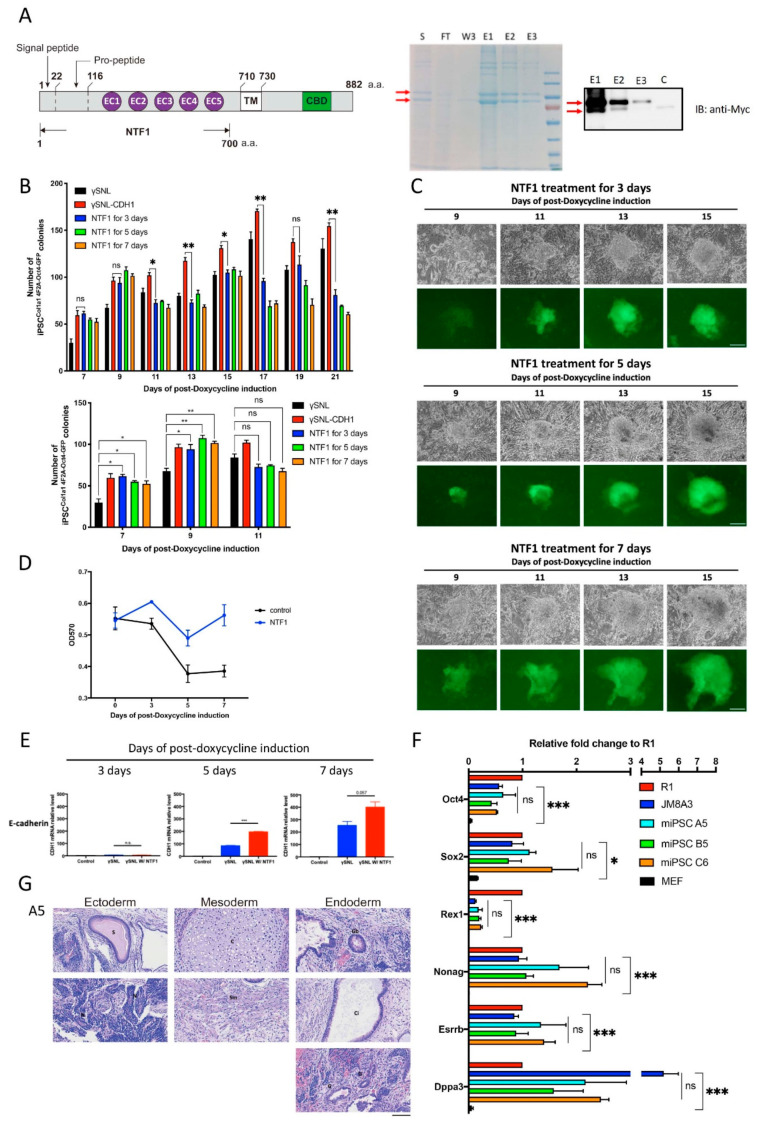
The effect of NTF1 recapitulates the SNL::CHD1-myc feeder adoption at the early stage of reprogramming. (**A**) Schematic display of the structure of NTF1 protein and purification of the recombinant NTF1 protein. The culture medium collected from the Freestyle 293 cells transfected with pCMV-CDH-NTF1-MYC-His was subjected to NTF1 protein purification. SDS PAGE and western blots were used to analyze the purified product. The red arrows indicate recombinant proteins with different degrees of posttranslational modification. (**B**) Quantitative analysis of Oct4-GFP-positive clones under different NTF1 treatments during reprogramming. The lower panel shows the data derived from the results of the 7th, 9th, and 11th days in the upper graph. Student’s t-test was applied for statistical evaluation (n = 3). (**C**) Time-lapse photographs of the Oct4-GFP-positive clones treated with various NTF1 exposure times. The scale bar denotes 50 µm. (**D**) The growth curve of the induced MEF^Col1a1 4F2A Oct4-GFP^ cells during the first seven days of iPSC induction. An MTT assay was used to quantify cell viability on days 0, 3, 5, and 7 after initiating the induction process. The data points represent the mean of triplicate results. (**E**) Comparative analysis of endogenous E-cadherin expression in NTF1-treated and untreated MEF cells at the early stage of reprogramming. Harvested cells were subjected to total RNA isolation for the RT-PCR-based gene expression analysis. Cells that did not undergo somatic reprogramming served as controls. (**F**) Quantitative RT-PCR of pluripotent markers in the above mentioned three iPSC clones compared with two standard ESCs, namely, R1 and JM8A3. (**G**) Teratoma formation analysis on the A5 iPSC clone to demonstrate its competency for formation of three germ layers. The scale bar denotes 100 µm. S: supernatant; FT: flow-through; W3: 3rd wash; E1: 1st elution; E2: 2nd elution; E3: 3rd elution. *, *p* < 0.05; **, *p* < 0.005; ***, *p* < 0.0005; ns, not significant.

Only the extracellular region (N-terminal region) of E-cadherin physically contacted the MEF^Col1a1 4F2A Oct4-GFP^ cells when SNL4-2 cells were used as the feeder cells. To verify that the observed effect is genuinely due to the exogenous CDH1 but not the other factors derived from the feeder cells, the SNL4-2 cells were replaced by the parental SNL cells plus the N-terminal region of E-cadherin peptide to determine whether a similar effect on facilitating the early stage of somatic reprogramming would be observed. For this purpose, a protein consisting of the N-terminal part of the truncated E-cadherin containing the entire extracellular domain fused with MYC- and His-tags at its C-terminus (hereafter called NTF1) was designed and purified from freestyle HEK293 cells. The predicted molecular weight of NTF1 was approximately 90 kDa (1-700 AAs, AAs 1-119 comprise the signal/pro-peptide; Figure 2A right panel). Two forms of NTF1, approximately 90 and 110 kDa, appeared on the SDS PAGE results, as shown in Figure 2A (right panel). Since these two recombinant proteins also appeared while probing with an anti-MYC antibody, they may represent the consequences of various degrees of posttranslational modification. Therefore, those two isoforms were pooled together and served as the NTF1 recombinant protein.

In the induction experiment, we used iSF medium supplemented with NTF1 (1 µg/mL), which was changed daily. Accordingly, 3, 5, and 7 days after the NTF1-treated groups were set up, and the progression of iPSC formation was observed on the 7th, 9th, and 11th days postdoxycycline induction. On the first two observation days, the number of iPSC^Col1a1 4F2A Oct4-GFP^ colonies with various NTF1 exposure times was indistinguishable among groups, but all formed significantly more iPSC clones than the control group (see the lower panel in Figure 2B). Such an effect of promoting reprogramming was observed until the 9th day postdoxycycline induction. Then, the number of the iPSC clones was consistent throughout the remaining induction course, whereas both the SNL and SNL4-2 control groups showed continuous increases. At the end of the 21-day induction course, the NTF1-treated groups only achieved half of the induction rate, as observed in the SNL4-2 group (see the upper panel in Figure 2B). Although the NTF1 treated groups did not generate iPSCs with a high induction rate, the quality of iPSCs with regard to colony morphology and OCT4-GFP intensity was comparable with that of the SNL and SNL4-2 groups (Figure 1C and Figure 2C). Thus, the same highly differentiated cell type is capable of adopting different reprogramming processes. In this case, the presence of NTF1 causes the MEF^Col1a1 4F2A Oct4-GFP^ cell to switch from undergoing the original stochastic process to the deterministic model of action.

Next, to determine how NTF1 may affect the selection of the reprogramming process at an early stage, we examined the proliferation of MEF^Col1a1 4F2A Oct4-GFP^ cells by MTT assays with or without the NTF1 treatment. As shown in Figure 2D, the NTF1-treated MEF cells increased in cell number during the first three days of treatment, decreased in cell number on the 5th day, and returned to the same proliferation rate on the 7th day. Although the control group shared a similar growth curve pattern, the growth rate was much slower than that of the NTF1-treated group. The sudden decrease in cell number on the 5th day post-NTF1 treatment was due to the death of the γ-irradiated feeder cells. Furthermore, we examined the expression of endogenous E-cadherin on days 3, 5, and 7 postdoxycycline induction. As shown in Figure 2E, a significant upregulation of endogenous E-cadherin was observed on the 5th day compared to the NTF1-untreated control. Although proliferation of MEF^Col1a1 4F2A Oct4-GFP^ cells occurred, we did not find a significant advancement in the kinetics of reprogramming. Thus, the elevation of endogenous E-cadherin expression along with the acceleration of proliferation indicated that the presence of NTF1 somehow elicits a different cell state of the MEF^Col1a1 4F2A Oct4-GFP^ subpopulation, allowing them to gain the capability to complete the reprogramming process.

### 3.3. NTF1-Treated MEF^Col1a1 4F2A Oct4-GFP^ Cells Acquire Pluripotency by Undergoing the Deterministic Route of Reprogramming

Cells that underwent iPSC^Col1a1 4F2A Oct4-GFP^ induction, along with those that were subjected to three different NTF1 treatment periods (i.e., 3-, 5- and 7-day treatments), were clonally isolated, cultivated, and expanded in R1 ESC medium. As shown in Figure S1A, Oct4-GFP was strongly expressed and maintained in clonally isolated iPSCs^Col1a1 4F2A Oct4-GFP^. Furthermore, to verify the state of pluripotency, a quantitative analysis of the pluripotency-associated genes, including Oct4, Sox, Rex1, Nanog, Esrrb2, and Dppa3, was conducted. All of these clones had a comparable expression level, as seen in two canonical mESC cell lines (i.e., R1 and JM8A3), whereas the MEF^Col1a1 4F2A Oct4-GFP^ cells served as a negative control to mark the basal levels before iPSC induction (See Figure 2F).

To further challenge their pluripotency, the three independent iPSC^Col1a1 4F2A Oct4-GFP^ clones isolated after 3-, 5-, and 7-day NTF1 treatments were subjected to teratoma formation assays to evaluate their capability to form germ layers. As shown in Figure 2G, the teratoma contains three germ layers with cell types derived from the 3-day NTF1 treatment. A comparable differentiation capacity was also observed in the iPSC^Col1a1 4F2A Oct4-GFP^ clones derived from the B5 clone (5-day NTF1 treatment) and the C6 clone (7-day NTF1 treatment) (Figure S1B). Thus, our NTF1-treated iPSC^Col1a1 4F2A Oct4-GFP^ clones acquired pluripotency and could be stably maintained in our culture system.

### 3.4. STAT3 and Erk1/2 Are Potential NTF1 Downstream Effectors during the Early Stage of Somatic Reprogramming

**Figure 3:**
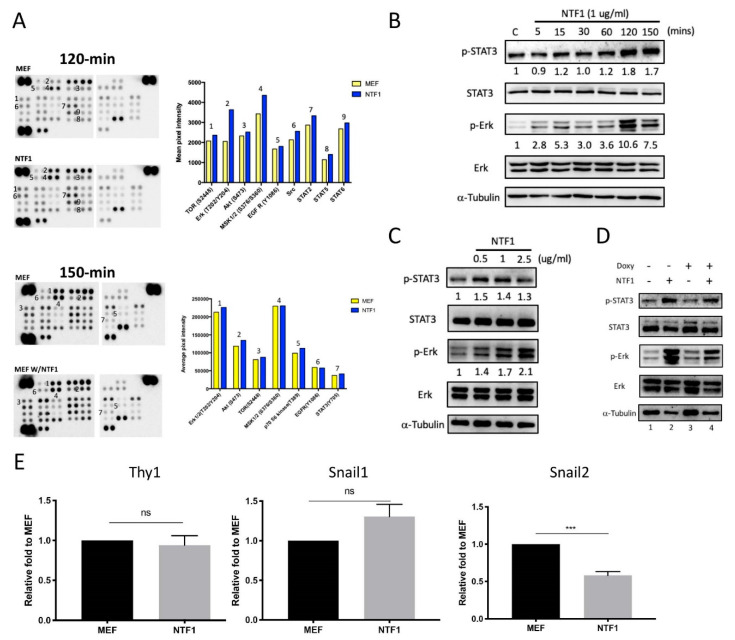
STAT3 and Erk1/2 regulated Snail2 expression are the potential signal transducers activated by NTF1 (**A**) Identification of the NTF1-mediated signal transducers through blotting of the phospho-kinase array. After 24-h serum starvation, MEF^Col1a1 4F2A Oct4-GFP^ cells treated with NTF1 for 120 and 150 min were subjected to the phospho-kinase array analysis, and untreated MEFs^Col1a1 4F2A Oct4-GFP^ served as the control. The numbers labeled on the bar graph correspond to the numbers on the blot images. The upper panel shows the 120 min NTF1 treatment, whereas the lower panel displays the 150 min treatment. (**B**) The time-dependent effect of NTF1 treatment on the phosphorylation of STAT3 and Erk1/2. After 24-h of serum starvation, MEF^Col1a1 4F2A Oct4-GFP^ cells were subjected to a time-course experiment via treatment with 1 µg/mL NTF1 for 5, 15, 30, 60, 120, or 150 min. The NTF1-untreated MEF^Col1a1 4F2A Oct4-GFP^ cells served as the control. In the 120 min NTF1 treatment group, both phosphorylated STAT3 and Erk1/2 signals reached a plateau on the blot. (**C**) The dose-dependent effect of NTF1 treatment. MEF^Col1a1 4F2A Oct4-GFP^ cells were subjected to different concentrations of NTF1 (0.5, 1, and 2.5 µg/mL). Both the phosphorylated STAT3 and Erk1/2 signals reached a plateau at 1 µg/mL after 120 min of NTF1 treatment. Untreated MEF^Col1a1 4F2A Oct4-GFP^ cells served as the control. (**D**) The effects of NTF1 treatment in the presence of doxycycline. After 24 h of serum starvation, MEF^Col1a1 4F2A Oct4-GFP^ cells were simultaneously subjected to NTF1 and doxycycline treatment for 120 min. (**E**) A comparative analysis of the MET-associated genes, including Snail1, Snail2, and Thy1, revealed that Snail2 is the downstream effector of NTF1 treatment targeted by STAT3 and Erk1/2. (*n* = 3). ***, *p* < 0.0005; ns, not significant.

To identify the possible downstream kinase participating in relaying the NTF1 signal, MEF^Col1a1 4F2A Oct4-GFP^ cells were treated with NTF1 for 120 and 150 min. The harvested cells lysate was blotted on the membrane of a phosphor-kinase array, and the untreated counterpart served as a control. Several kinases were identified by comparing the signal intensity between treated and untreated samples at two different time points. As shown in the upper panel of Figure 3A, the upregulated kinases included TOR, Erk1/2, MSK1/2, STAT2, and STAT5 from the 120 min treatment. Activation of STAT3 was detected for the 150 min treatment condition (Figure 3A lower panel). Because prior studies demonstrated the involvement of STAT3 and Erk1/2 in ESC/iPSC self-renewal and pluripotency maintenance, we therefore selected both STAT3 and Erk1/2 for further studies regarding their roles at the onset of somatic reprogramming.

In addition to detecting phosphorylation of STAT3 and Erk1/2 in the phosphor-kinase array, the time course experiments also showed consistent results on western blots. As shown in Figure 3B, MEF^Col1a1 4F2A Oct4-GFP^ cells were treated with NTF1 for 5, 15, 30, 60, and 120 min. The elevated phosphorylation level of STAT3 and Erk1/2 followed the increasing exposure time to NTF1 and reached the highest phosphorylation level at the 120 min time point. Next, to uncover the dosage effects of extraneous E-cadherin on the phosphorylation of STAT3 and Erk1/2, MEF^Col1a1 4F2A Oct4-GFP^ cells were subjected to 120 min treatment with 0.5, 1, or 2.5 µg/mL of NTF1. Phosphorylation of both STAT3 and Erk1/2 reached a plateau at a concentration of 1 µg/mL (Figure 3C).

During the iPSC induction process, the effects of extraneous E-cadherin combined with the presence of doxycycline to induce the expression of Yamanaka factors. The presence of doxycycline and, therefore, the subsequent appearance of Yamanaka factors may activate STAT3 and Erk1/2 instead. To exclude this possibility, we again evaluated the phosphorylation level of STAT3 and Erk1/2 in the presence or absence of doxycycline. As shown in Figure 3D, the effect of doxycycline is negligible. Collectively, STAT3 and Erk1/2 were the two main, or possibly only, downstream targets of extraneous E-cadherin. To further identify the downstream effectors of STAT3 and Erk1/2 that participate in the early stage of reprogramming, we examined the expression of MET-associated genes because we observed the upregulation of endogenous E-cadherin after the NTF1 treatment (see Figure 2E). Accordingly, the expression of Snail1, Snail2, and Thy1 in MEF^Col1a1 4F2A Oct4-GFP^ cells after NTF1 treatment was examined. As shown in Figure 3E, significant downregulation of gene expression was observed for only Snail2 and not the other two genes compared with the NTF1-untreated control. Consistent with previous reports, Snail2 is a direct downstream target gene of STAT3 and epigenetically represses E-cadherin [16,17].

### 3.5. NTF1 Activates STAT3 and Erk1/2 via LIFR/gp130 and EGFR/ErbB2 During the Early Stage of Somatic Reprogramming

**Figure 4:**
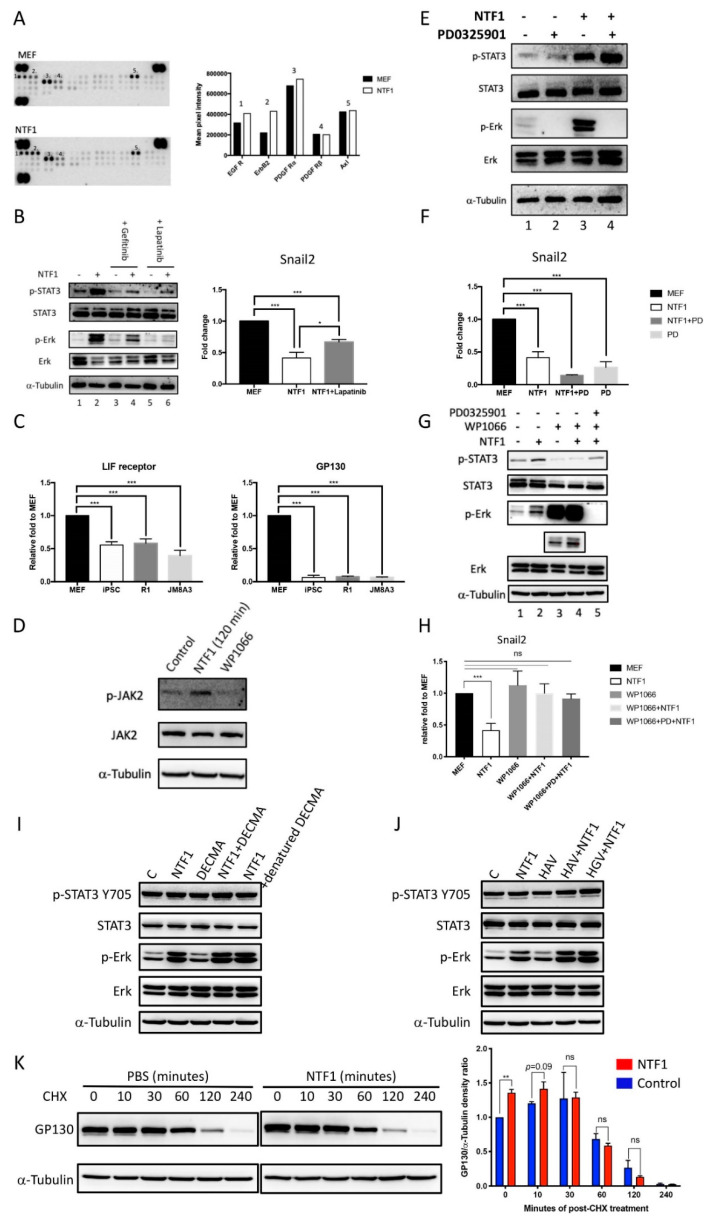
NTF1 may stabilize the LIFR/gp130 complex to transduce the STAT3-mediated signal cascade (**A**) Use of a receptor tyrosine kinase array revealed the potential NTF1-interacting candidates on the MEF^Col1a1 4F2A Oct4-GFP^ membrane. The numbers on the bar graph correspond to the numbers on the blot images. (**B**) Examination of the signals involved in the NTF1-mediated signal transduction pathways using Gefitinib and Lapatinib, which are specific inhibitors of EGFR and EGFR/ERbB2, respectively. Using the same treatments, the harvested total RNA of the treated cells was subjected to quantitative RT-PCR analysis with a Snail2-specific primer set. (**C**) A comparative RT-PCR analysis of LIFR and gp130 in MEF^Col1a1 4F2A Oct4-GFP^, iPSC^Col1a1 4F2A Oct4-GFP^, R1 ESC, and JM8A3 ESC cells. (**D**) Treatment with NTF1 also activates JAK2. MEF^Col1a1 4F2A Oct4-GFP^ cells treated with NTF1 or WP1066 for 120 min were subjected to phosphorylation analysis of Jak2, and untreated MEFs served as the control. (**E**,**F**) A synergistic effect on repression of Snail2 expression was observed in the presence of both PD0325901 and NTF1. Using the same treatments, the counterpart experiment was subjected to quantitative RT-PCR analysis with a Snail2-specific primer set. (**G**,**H**) Inactivation of STAT3 abolishes the repression of Snail2 expression. MEF^Col1a1 4F2A Oct4-GFP^ cells were subjected to 24-h serum starvation before treatment with NTF1, WP1066, NTF1+WP1066, or NTF1+WP1066+PD0325901. Both cell lysates and total RNA were extracted and subjected to western blotting and RT-PCR analyses, respectively. (**I**,**J**) The effect of the DECMA-1 antibody and HAV and HGV peptides in the NTF1-treated experiments. The working concentrations of DECMA-1 (ThermoFisher Scientific, Waltham, MA, USA; 14-3249-82) and the HAV and HGV peptides were 5 µg/mL, 5 mM, and 5 mM, respectively. (**K**) NTF1 stabilized gp130 in the first 10 min treatment. In the left panel, cycloheximide (CHX) was applied to MEF cells following treatment with NTF1 or PBS. A time-course experiment was performed to observe the change in the gp130 expression level at 0, 10, 30, 60, 120, and 240 min after applying the treatments. The working concentration of CHX was 50 mM. The right panel displays the quantitative result of western blot signals. All groups were normalized to the 0 min treatment in PBS. In each group, the normalized gp130 value was divided by the amount of α-tubulin. Bars represent the mean ratios of each group, and the experiment was performed in duplicate. “+” and “-” denote that the factor was present or absent in the medium, respectively. The working concentrations of NTF1, WP1066, and PD0325901 were 1 µg/mL, 10 µM and 1 µM, respectively. *, *p* ≤ 0.05; **, *p* ≤ 0.005; ***, *p* ≤ 0.0005; ns, not significant.

To identify specific membrane receptors that interact with NTF1 and facilitate early somatic reprogramming, we used a receptor tyrosine kinase (RTK) array to screen potential NTF1-interacting candidates (R&D systems, Minneapolis, MN, USA). Similar to the prior phosphor-kinase array, the 120 min NTF1-treated MEF^Col1a1 4F2A Oct4-GFP^ cell lysates were subjected to RTK array analysis. The quantified intensity of binding activity from the RTK array revealed a few potential membrane receptors with significant differences between the NTF1-treated and untreated groups, including EGFR and ErbB2 (Figure 4A; dots labeled No. 1 and 2, respectively).

To determine whether the upregulation of STAT3 and Erk1/2 activity by NTF1 occurs via EGFR and ErbB2, we used Gefitinib and Lapatinib in the following experiments. Gefitinib is a specific inhibitor of EGFR, whereas Lapatinib is an inhibitor of EGFR/ERbB2 [18]. In the absence of NTF1, either Gefitinib or Lapatinib only slightly decreased the pSTAT3 level but did not affect that of pErk1/2 (Figure 4B, comparing both lanes 3 and 5 with lane 1). In the presence of NTF1, both inhibitors suppressed the elevation of both pSTAT3 and pErk1/2 levels (Figure 4B, comparing both lanes 4 and 6 with lane 2). Interestingly, Lapatinib alone could completely block the activation of Erk1/2 by NTF1 (Figure 4B, lane 6). Although the presence of EGFR/ErbB2 inhibitors indirectly affects the phosphorylation level of STAT3, primary STAT3 activation, however, may occur through the canonical LIFR/gp130 route. As shown in Figure 4C, the abundance of LIFR and gp130 in MEF^Col1a1 4F2A Oct4-GFP^ cells was higher than that in iPSCs and mESCs. Additionally, the 120 min NTF1 treatment induced activation of JAK2, indicating involvement of the LIFR/gp130 pathway in STAT3 activation (Figure 4D).

In summary, NTF1 activates Erk1/2 through EGFR and ErbB2 in the MEF^Col1a1 4F2A Oct4-GFP^ context. Lapatinib, a specific EGFR/ErbB2 inhibitor, can completely suppress the activation of Erk1/2 and partially influence the increase in phosphorylation of STAT3 induced by NTF1 treatment. In addition to transducing the effect of NTF1 through EGFR/ErbB2, activation of JAK2 activity was also observed, which is responsible for the activation of STAT3. Therefore, the NTF1 promoting effect observed at the early stage of somatic reprogramming may occur through the EGFR/ErbB2 and LIFR/gp130 signal transduction pathway axis.

### 3.6. Phosphorylated STAT3, but not Erk1/2, Is a Pivotal Modulator That Promotes Somatic Reprogramming at the Early Stage of Somatic Reprogramming

The above results showed that NTF1 induces activation of STAT3 and Erk1/2 through the EGFR/ErbB2 and LIFR/gp130 routes. However, how activation of STAT3 and Erk1/2 facilitates the early stage of iPSC induction is still unclear. As previously mentioned, this effect occurs at approximately the 7–9th day postdoxycycline treatment, which is similar to the occurrence of the MET. The observed decrease and increase of Snail2 and E-cadherin, respectively, further prompted us to examine the interplay between STAT3 and Erk1/2 at the onset of somatic reprogramming.

To determine whether the downregulation of Snail2 was associated with the activation of STAT3 and Erk1/2, WP1066 and PD0325901, specific small molecule inhibitors, Jak2 kinase and pErk1/2, respectively, were used to validate the relationship between the activation of STAT3 and Erk1/2 and the expression of Snail2. As shown in Figure 4E, PD0325901 completely suppressed the basal level of pErk1/2, even in the presence of NTF1. Additionally, a significant elevation of pSTAT3 was observed when both PD0325901 and NTF1 were included. Under these conditions, the expression level of Snail2 was decreased when pSTAT3 was increased; a reduction of Snail2 expression was detected when MEF^Col1a1 4F2A Oct4-GFP^ cells were treated with either NTF1 or PD0325901, whereas a further synergistic effect on the suppression of Snail2 expression was observed in the presence of both PD0325901 and NTF1 (Figure 4F).

Next, we investigated the involvement of LIFR/gp130 triggered by NTF1 on the activation of STAT3. As WP1066 is a specific inhibitor of Jak2, the inclusion of WP1066 strongly inhibited the activation of STAT3 induced by NTF1 (see Figure 4G, lane 2 and 4). The same conditions were tested by the quantitative RT-PCR to examine the expression level of Snail2. The results demonstrated that the downregulation of Snail2 was restored by WP1066 (see Figure 4H). Treatment of MEF^Col1a1 4F2A Oct4-GFP^ cells with WP1066, WP1066+NTF1, or WP1066+NTF1+PD0325901 did not produce any significant differential effect on Snail2 expression. To further demonstrate the relevance of Erk1/2 activation and Snail2 expression, MEF^Col1a1 4F2A Oct4-GFP^ cells were subjected to treatment with Lapatinib, and Snail2 expression was measured. As expected, the downregulation of Snail2 expression was partially restored in the presence of both NTF1 and Lapatinib (Figure 4B, right panel).

Taken together, the application of specific molecule inhibitors (i.e., WP1066, PD0325901, and Lapatinib) helped to uncover the potential NTF1-mediated signal transduction at the early stage of somatic reprogramming. The blockage of pErk1/2 by PD0325901 in the presence of NTF1 had a synergistic effect on suppressing Snail2 expression, whereas suppression of pSTAT3 by WP1066 completely abolished the downregulation of Snail2, even in the presence of NTF1. These results are consistent with the idea that NTF1 acts as an upstream effector to modulate the cell fate of MEF^Col1a1 4F2A Oct4-GFP^ cells at the early stage of somatic reprogramming. The observation of the dramatic activation of Erk1/2 and the repression of pSTAT3 mediated by WP1066 is consistent with previous reports regarding the antagonistic effect between STAT3 and Erk1/2 (Figure 4G, lane 3 and 4; Figure 4E, lane 2 and 4) [19,20]. Although NTF1 promotes the activation of both STAT3 and Erk1/2 through LIFR/gp130 and EGFR/ErbB2, only activation of STAT3 is needed to suppress the expression of Snail2 and, in turn, facilitate the change in MEF cell fate. In contrast, activation of Erk1/2 has an adverse effect by promoting Snail2 expression. Together, the deactivation of pErk1/2 by PD0325901 can further downregulate Snail2 in the presence of NTF1. Thus, facilitation of the change in cell fate at the early stage of somatic reprogramming by NTF1 mainly occurs through STAT3 activation.

### 3.7. The Effect of NTF1 May Occur through Stabilizing the LIFR/gp130 to Transduce the STAT3-Mediated Signal Cascade

E-cadherin-mediated cell adhesion and signaling occur through the homophilic interaction between adjacent cells. Although MEF cells do not express E-cadherin, the extracellular domain of E-cadherin may exert other mechanisms that trigger the LIF/gp130- and EGFR/ErbB2-mediated signal cascade. Therefore, we used DECAM-1 (an E-cadherin ectodomain-specific mAb) and an HAV/HGV inhibitory peptide (SHAVSA/ SHGVSA) to interfere with the E-cadherin-mediated homophilic interaction. As shown in Figure 4I,J, the inclusion of either DECAM-1 or the HAV inhibitory peptide with NTF1 did not inhibit the NTF1-mediated activation of STAT3 and Erk1/2. Additionally, the presence of the HAV peptide alone did not trigger the event, as observed with NTF1 treatment. Thus, the mechanism of NTF1-mediated activation of STAT3 and Erk1/2 signaling is unrelated to the homophilic interaction domain of E-cadherin.

Alternatively, such signaling triggering by the extracellular domain of E-cadherin may occur through a complex with receptors. As prior reports demonstrated, the extracellular domain of E-cadherin appeared to stabilize the heterodimerization of the ErbB2 (HER2)/HER3 complex to induce the Erk-mediated pathway [21]. In that regard, we tested whether the presence of NTF1 also stabilizes the LIF/gp130 receptor complex. Therefore, we used cycloheximide treatment to determine whether the presence of NTF1 can prevent the internalization and degradation of gp130 in a time-dependent manner. As shown in Figure 4K, the presence of NTF1 did stabilize gp130 and prevent it from being degraded during the first 10 min interval. These results are consistent with a previous report that showed that the extracellular domain of E-cadherin forms a complex with the LIFR/gp130 receptor to promote signaling in mESCs {22}. Taken together, the presence of NTF1 may stabilize the receptors to induce their signaling processes, which does not require a homophilic interaction.

### 3.8. Deactivation of pErk1/2 by PD0325901 at the Early Stage of Somatic Reprogramming Further Promotes iPSCCol1a1 4F2A Oct4-GFP Formation but Returns to the Stochastic Process

The above findings have established that extraneous E-cadherin-mediated early-stage somatic reprogramming occurs through the signal transduction axes of EGFR/ErbB2 and LIFR/gp130 and their downstream effectors, STAT3 and Erk1/2. Activation of STAT3, along with deactivation of Erk1/2, can promote the suppression of Snail2 and potentially overcome the MET barrier. Accordingly, the inclusion of PD0325901 along with NTF1 can further benefit the early stage of somatic reprogramming.

**Figure 5:**
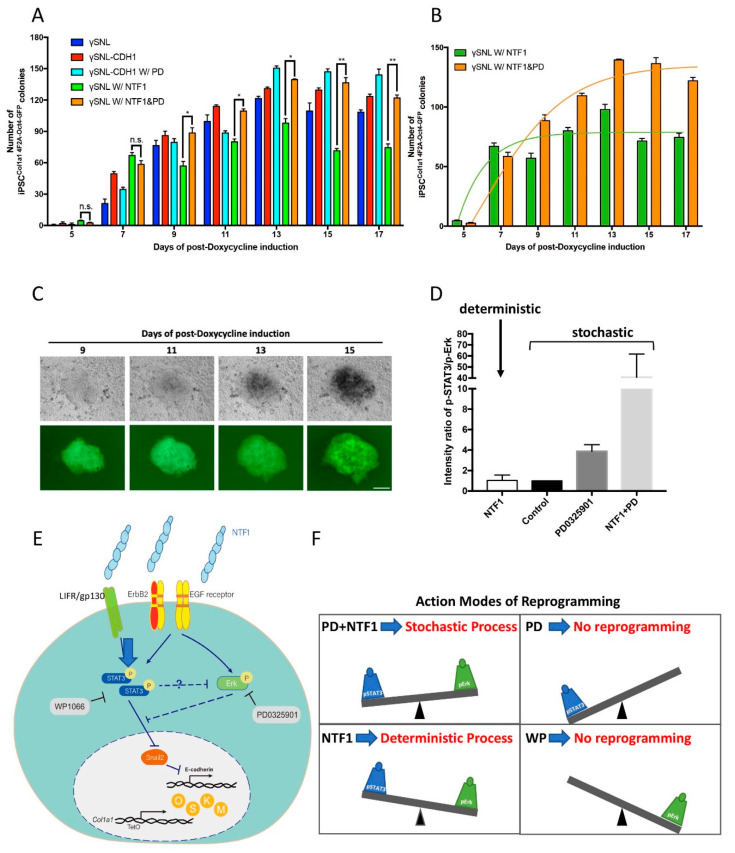
The differential effects of NTF1 and PD0325901 on the choice of programming process at the onset of iPSC induction (**A**) The quantitative analysis of iPSC colony formation under various treatments. The number of the OCT4-GFP-positive clones is indistinguishable between MEF cells treated with NTF1+PD0325901 and those treated with NTF1 alone on the 7th day postdoxycycline induction. However, the NTF1 alone condition maintained the OCT4-GFP-positive clone number observed on the 7th day throughout the entire course of induction (green bar). In contrast, the NTF1+PD0325901-treated group showed an increase the OCT4-GFP-positive clone number until the end of induction (orange bar). (**B**) The curve-fitting data of the NTF1 (green) and NTF1+PD (orange) groups extracted from panel A. The NTF1-treated group (green) represents the deterministic process, whereas the NTF1+PD group (orange) shows the stochastic model. (**C**) Representative time-lapse photographs of iPSC^Col1a1 4F2A Oct4-GFP^ clone formation induced by NTF1+PD0325901. The series of photographs was obtained from the same clone from the 9th to 15th day postdoxycycline induction. The scale bar denotes 50 µm. (**D**) The selection of a reprogramming process depends on the pSTAT3 vs. pErk1/2 ratio. In the presence of NTF1, the pSTAT3/pErk1/2 ratio creates a unique cell state and a threshold where it engages the deterministic process during reprogramming. If the ratio is higher than the NTF1-established ratio, reprogramming will occur through the stochastic route instead. (**E**) A proposed mechanism addresses the selection of the somatic reprogramming mode. The current model represents an example in which the surrounding microenvironment imposed upon MEF^Col1a1 4F2A Oct4-GFP^ cells changes the cell fate at the onset of iPSC formation. Extraneous E-cadherin transduces the signal through EGFR/ErbB2 and LIFR/gp130 resulting in activation of the downstream effectors STAT3 and Erk1/2. As the activities of pSTAT3 and pErk1/2 counteract each other, the net output through the respective down- and upregulation of Snail2 and E-cadherin overcomes the MET barrier. In this scenario, pErk acts as a negative regulator on the entry of reprogramming. Accordingly, the balance between STAT3 and Erk1/2 activity is thus essential in setting the stage for cell fate at the entry point of somatic reprogramming. (**F**) In addition to controlling the entry, the balance between STAT3 and Erk1/2 activity also regulates the choice of entry model, where either the deterministic or stochastic process could be chosen at the onset of reprogramming. The presence of NTF1 in the surrounding environment alters the ratio of pSTAT3 and pErk activity, resulting in adoption of the deterministic model. In contrast, the increment of STAT3 activity, along with the deactivation of Erk, engages the stochastic model in the presence of NTF1+PD0325901 for the first three days of induction. Previous reports have supported that either deterministic or stochastic processes could be adopted during reprogramming depending on the source of the cell type. In the present study, our evidence supports that different modes of operation can act on the same MEF cells and are interchangeable via modulation of the STAT3 and Erk activity. n = 3. PD: PD0325901; WP: WP1066. *, *p* < 0.05; **, *p* < 0.005; ns, not significant.

To correlate the above molecular findings with the changes in cell fate during iPSC induction, we included both PD0325901 and NTF1 during the first three days of iPSC induction. Both the NTF1-only and the NTF1+PD0325901 groups displayed a better induction rate than the parental SNL line at the early stage (Figure 5A, dark blue and orange bars). Intriguingly, the group treated with PD0324901+NTF1 for three days not only exhibited facilitated iPSC formation at an early stage but also displayed a comparable effect to that observed in the SNL::CDH1-MYC group at the later stage of somatic reprogramming (Figure 5A, orange and red bars). The colonies that appeared in all of the tested groups displayed a typical ESC-like morphology, consisting of a dome-shaped morphology with a reflective edge (see Figure 5C). Furthermore, the PD0325901-treated group with SNL::CDH1-MYC feeder cells showed a slight increase in the efficiency of the induction rate compared to the SNL::CDH1-MYC feeder only group (Figure 5A, red and light blue bars). In summary, the presence of PD0325901 for the first three days in our iPSC induction system further advanced the process of somatic reprogramming, which is consistent with our previously mentioned regulatory network mediated by the NTF1 at the early stage of iPSC formation. However, the presence of PD0325901 for three days changed the NTF1-dependent deterministic model to the stochastic process (see Figure 5B).

### 3.9. The Potential Mechanism of the Action for Selecting the Stochastic or Deterministic Process at the Early Stage of Somatic Reprogramming

Based on the results of the present study, a signal-transducing cascade by extraneous E-cadherin was proposed. The EGFR/ErbB2 and LIFR/gp130 receptors detect the presence of extraneous E-cadherin in the surroundings. Following activation of LIFR/gp130 and EGFR/ ErbB2 receptors, the relayed signals activate STAT3 and Erk1/2 through their phosphorylation cascades. Although these two kinases play different roles in the downstream events, the activated STAT3 downregulates Snail2 and facilitates the process of epigenetic remodeling (e.g., mesenchymal-epithelial-transition; MET). In contrast, the phosphorylated Erk1/2 activates the expression of Snail2 (see Figure 4H). Consistent with previous reports, we observed that activated STAT3 directly or indirectly antagonizes the phosphorylation of Erk1/2 [19,20] (see Figure 4G). Thus, activated Erk1/2 and STAT3, via NTF1, mutually antagonize each other by establishing a unique cell state in the context of MEF cells. When a LIFR/gp130-JAK2-STAT3 dominant pathway is created, this activation facilitates a subpopulation of MEFs to surpass the MET barrier and permits this subpopulation of cells to reach the pluripotent state. Such an antagonizing effect is even more evident if the expression of Snail2 is further reduced in the presence of PD0325901 (Figure 4F). Although an apparent increment of iPSC clones was observed while MEF cells were treated with NTF1+PD0325901 for the first 3 days of induction, the route of reprogramming was switched to the stochastic process instead (Figure 5A,B).

Collectively, our findings illustrate that the presence of extraneous E-cadherin activates both the LIFR/gp130-STAT3 and EGFR/ErbB2-Erk1/2 transduction pathways through stabilizing corresponding receptors in MEF^Col1a1 4F2A Oct4-GFP^ cells. The sum of the differential output of these two pathways determines the choice of the reprogramming process (Figure 5E). In this scenario, the refractory activity derived from pErk1/2 also participates in the NTF1-mediated somatic reprogramming. Thus, suppression of pErk1/2 is not only crucial for maintaining the state of pluripotency but also promotes the progression of early-stage somatic reprogramming [3]. Unlike the self-sustained pluripotent state, the requirement for Erk1/2 activity during the process of somatic reprogramming is essential. Since ERK1/2 positively regulates cell proliferation according to its expression level, the presence of PD0325901 during the entire course of reprogramming suppresses MEF cell proliferation if iPSC formation does not occur (data not shown) [22].

Additionally, the present study also first showed that OSKM-mediated reprogramming can adopt either stochastic or deterministic processes in the MEF context. Such a decision regarding the action mode may depend on the ratio of pSTAT3 and pErk1/2 at the onset of iPSC induction. In the presence of NTF1, both activated STAT3 and Erk1/2 establish a different ratio, favoring the deterministic process. While such a ratio is higher than that produced by the NTF1-mediated effect, which includes treatment with of NTF1+PD0325901 and PD0325901, the process will remain within the stochastic model (Figure 5D,F). Thus, the activity of pSTAT3 and pErk1/2 may define the different cell states of MEF cells by responding to external signals.

## 4. Discussion

In the present study, we attempted to understand the composition of and how the surrounding microenvironment participates in the molecular events of somatic reprogramming. We found that providing the extracellular domain of the CDH1 (NTF1) recombinant protein will induce the EGFR/ErbB2 and LIFR/gp130 signal transduction pathways to facilitate the early stage of somatic reprogramming, potentially through overcoming the MET barrier. The NTF1-mediated crosstalk establishes a unique output of the pSTAT3 and pErk1/2 ratio, and therefore, a selected subset of cells undergoes somatic reprogramming by adopting the deterministic process. Applying specific molecular inhibitors to target the activity of either pSTAT3 or pErk1/2 enables conversion of the action mode between the stochastic and deterministic models. Thus, the selection of the reprogramming process may depend on the cell state with regard to balanced pSTAT3 and pErk1/2 activity.

The traditional approach using virus-based iPSC production generates heterogeneity resulting from the introduction of various copy numbers of Yamanaka factors into cells. Such a heterogeneous population may obscure any potential effects imposed by the surrounding microenvironment. Therefore, we used MEF cells from homozygous Col1a1-4F2A-Oct4-GFP mice containing two copies of Yamanaka factors in the Col1a1 loci as a model of somatic reprogramming to uncover the effect of the microenvironment [23]. First, we manipulated the microenvironment by engineering the feeder cells to express E-cadherin (SNL::CDH1) and identified its effect on promoting iPSC formation. Furthermore, treatment with NTF1 recapitulated the effect of SNL::CDH1, which suggests that the components of the surrounding environment affect the process of somatic reprogramming.

Prior studies have demonstrated that overexpression or chemical intervention leading to upregulation of E-cadherin in cell reprogramming could replace the requirement for exogenous OCT4 during the process of somatic reprogramming [14,24]. Such manipulations may either directly or indirectly affect the transcriptional circuit for pluripotency. In the current study, the presence of extracellular NTF1 facilitated early-stage somatic reprogramming events, which were mediated mainly through the EGFR/ErbB2 and LIFR/GP130 signal transduction pathways, but these may not be the only routes. Moreover, the presence of NTF1 regulated the increase and decrease in endogenous E-cadherin and Snail2, respectively, indicating that NTF1 facilitates surpassing the MET barrier. The potential effect derived from NTF1 entering the cell cannot be excluded. Our data, however, demonstrated that both pSTAT3 and pErk1/2 are epistatic to NTF1 treatment regarding the expression of Snail2. Thus, the results indicate that the impact of NTF1 entry into cells is neglectable or nonexistent.

In addition to the pathways mentioned above, other routes taken by NTF1 can also activate STAT3 and Erk1/2. One possible course is via the homophilic interaction in trans with endogenous E-cadherin during reprogramming of MEF cells. Since our data indicated that endogenous E-cadherin in MEF^Col1a1-4F2A-Oct4-GFP^ cells is not detectable, a transduction signal that occurs via a homophilic interaction in trans is therefore unlikely (data not shown). Alternatively, the extraneous NTF1 may stabilize the LIFR/gp130 and EGFR/ErbB2 receptor complex to activate the STAT3- and Erk1/2-mediated epigenomic remodeling, respectively. Previous reports showed that extracellular E-cadherin stabilizes the LIFR/gp130 and ErbB2 (HER2)/HER3 complexes to activate STAT3 and Erk1/2 with regard to maintaining the pluripotency of ES cells and is associated with the progression of human cancer, respectively [21,25]. Such a signal transduction promoting mechanism also seems to be the case in the present study; the results of cycloheximide and inhibitory peptide treatments suggest a role of E-cadherin in receptor complex stabilization.

It is noteworthy that the effect of NTF1 on promoting the onset of somatic reprogramming was observed in treatments lasting less than 3 days. The critical window for NTF1 treatment to achieve such a prominent effect is thus within the first 72 h of programming. In the control groups using either parental SNL or the SNL:: E-cadherin feeder cells, an increase in the number of iPSC clones along with the progression of iPSC formation was observed. However, the resulting iPSC colony number of the NTF1-treated group seems to have been determined as early as the first seven days of reprogramming. Thus, the presence of NTF1 may allow only a unique MEF^Col1a1-4F2A-Oct4-GFP^ subpopulation to respond to the Yamanaka factors, while the remaining cells fail to further response to the Yamanaka factors. Interestingly, the sense of the remodeling factors is restored once the deactivation of Erk1/2 is achieved; in the presence of NTF1 and the Erk1/2-specific inhibitor (PD0325901), the typical stochastic model with a gradual incrementing iPSC clone number pattern reappeared. Therefore, transient treatment with NTF1 for less than three days favors somatic reprogramming via the deterministic process, whereas the γSNL, γSNL::E-cadherin, γSNL+NTF1+PD0325901, and γSNL+PD0325901-treated groups retained a stochastic process.

Because NTF1 activates both STAT3 and Erk1/2 which participate in the early somatic reprogramming in an antagonizing fashion, we thus posit that the entry permit of somatic reprogramming may correlate with the ratio of pSTAT3 and pErk1/2 activity, which in turn leads to adoption of a different model [26]. In the presence of NTF1, the pSTAT3/pErk1/2 ratio may result in a high threshold, permitting only privileged cells to proceed to reprogramming. As such, NTF1 treatment only allows a subpopulation of the MEF^Col1a1 4F2A Oct4-GFP^ cells to overcome the MET barrier in a synchronized manner. When Erk1/2 is deactivated by either PD0325901 alone or PD0325901+NTF1, the entry threshold is reduced via a higher pSTAT3/pErk1/2 ratio, which permits most cells to gradually alter their epigenetic configuration in a stochastic manner during iPSC formation.

Similar to previous reports, the stochastic process is predominantly associated with OSKM-mediated somatic reprogramming [5]. However, manipulating genetic drivers or isolating subpopulations of donor cells may alter this process by changing its reprogramming routes or activities [5]. Here, we provide the first evidence that the mode of action in somatic reprogramming can be changed in the same MEF population. In the presence of NTF1, the originally adopted stochastic course is altered to the deterministic model during the process of pluripotency acquisition. The choice of such a course potentially relies on the pSTAT3/pErk1/2 ratio at the early stage of reprogramming. Therefore, the surrounding microenvironment can affect the process chosen at the early stage of somatic reprogramming.

## Supplementary Materials

The following are available online at https://www.mdpi.com/2073-4409/10/2/284/s1, Figure S1: The NTF1-mediated somatic reprogramming of the MEF Col1a14F2AOct4-GFPacquires pluripotency, Table S1: Quantitative PCR primer list.

## Abbreviations

Induced Pluripotent Stem Cell (iPSC), The Col1a1 4F2A Oct4-GFP triple transgenic mouse (transgenic mouse contains extraneous Oct4, Sox2, Klf4 & cMyc genes inserted at the Collagen 1a1 locus). OSKM transgene (the coding of Oct4, Sox2, Klf4 & cMyc genes linked by the 2A sequences), SNL (a mouse fibroblast STO cell line transformed with neomycin resistance and murine LIF genes), MEF (mouse embryonic fibroblast).
