# Supplementary material for: Extraneous E-Cadherin Engages the Deterministic Process of Somatic Reprogramming through Modulating STAT3 and Erk1/2 Activity
Source: Cells. 2021 Jan 31;10(2):284. doi: 10.3390/cells10020284 (PMC7912071; doi:10.3390/cells10020284)
Supplement: Supplementary file 1 [file cells-10-00284-s001.pdf]

Supplement Figures

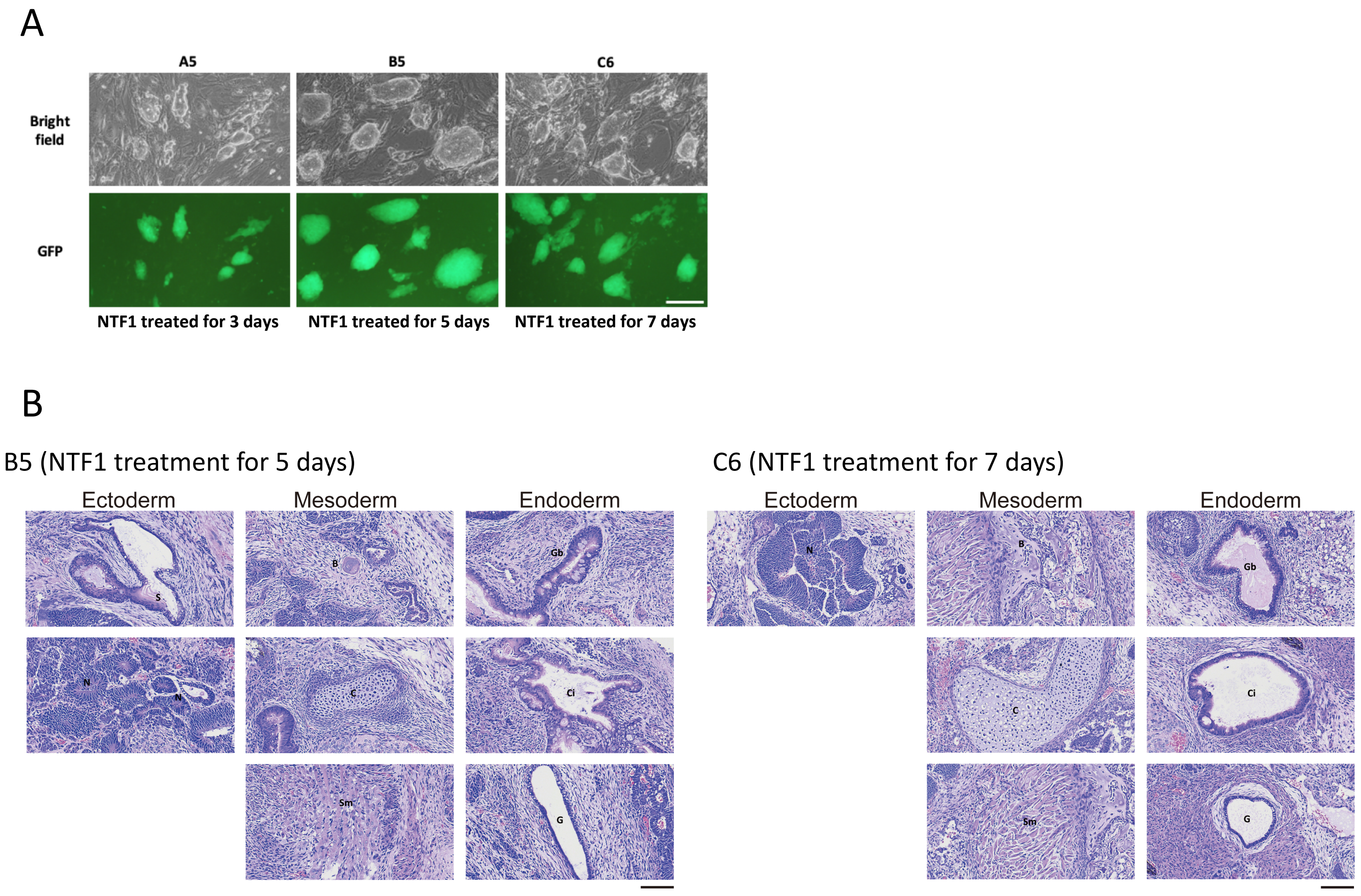

Figure S1. The NTF1-mediated somatic reprogramming of the MEF$^{Col1a1\ 4F2A\ Oct4\text{-}GFP}$ acquires pluripotency

(A) Representative clonally isolated iPSCs from three-day (A5), five-day (B5), and seven-day (C6) NTF1 treatments. The iPSC$^{Col1a1\ 4F2A\ Oct4\text{-}GFP}$ clones were cultured and maintained with γDR4 feeder cells in R1 ESC medium. (B) Teratoma formation assays of the iPSC B5 and C6 clones. S: squamous epithelium; N: neuronal rosette; B: bone; C: cartilage; Sm: skeletal muscle; Gb: Gut epithelium with goblet cells; Ci: ciliated respiratory-like epithelium; G: glands. Scale bar denotes 100 μm.

Supplement Tables

Table S1. Quantitative PCR primer list

Genes	Forward	Reverse
Snail1	5'-TGT GTC TGC ACG ACC TGT G-3'	5'-AGT GGG AGC AGG AGA ATG G-3'
Snail2	5'-ACA CAT TGC CTT GTG TCT GC-3'	5'-GCC CTC AGG TTT GAT CTG TC-3'
CDH1	5'-AAC CCA AGC ACG TAT CAG GG-3'	5'-GAG TGT TGG GGG CAT CAT CA-3'
Thy1	5'-TCG CTC TCC TGC TCT CAG TC-3'	5'-TTA TTC TCA TGG CGG CAG TC-3'
Oct4	5'-ACA TCG CCA ATC AGC TTG G-3'	5'-AGA ACC ATA CTC GAA CCA CAT CC-3'
Sox2	5'-ACA GAT GCA ACC GAT GCA CC-3'	5'-TGG AGT TGT ACT GCA GGG CG-3'
Nanog	5'-CCA GGT TCC TTC CTT CTT CC-3'	5'-GGT GAG ATG GCT CAG TGG AT-3'
Rex1	5'-ACG GAT ACC TAG AGT GCA TCA TAC G-3'	5'-TTG CTC CAC TTC CTC CAA GCT-3'
Esrrb	5'-TTT CTG GAA CCC ATG GAG AG-3'	5'-AGC CAG CAC CTC CTT CTA CA-3'
Dppa3	5'-CTG AAA GAC CCT ATA GCA AAG A-3'	5'-CTC ACT GTC CCG TTC AAA CTC A-3'
LIF r	5'-AGA AGA ACT GGC TCC CAT TG-3'	5'-GGA TGT CGT CCC ATT TCA CT-3'
GP130	5'-ATA GTC GTG CCT GTG TGC TTA-3'	5'-GGT GAC CAC TGG GCA ATA TG-3'
GAPDH	5'-GAA AGC TGT GGC GTG AT-3'	5'-TCC ACG ACG GAC ACA TT-3'
